# The comparison of the effectiveness and safety of drospirone ethinyl estradiol and ethinyl estradiol cyproterone in the treatment of polycystic ovarian syndrome: A protocol for systematic review and meta-analysis

**DOI:** 10.1097/MD.0000000000023811

**Published:** 2020-12-18

**Authors:** Zhimin Liu, Ying Song, Yuanfang Xu, Jing Wang, Hongyuan Hu, Yingchun Weng

**Affiliations:** a Department of Reproductive Medicine, Wenchang People's Hospital, No. 42 Wenqing Avenue, Wencheng Town, Wenchang City; b Department of Gynecology Clinic, Hainan Modern Women & Infants Hospital, NO. 16 Jinyu East Road, Longhua District, Haikou City; c Department of Gynecology, People's Hospital of Wanning, No. 1 Huanshi 3rd East Road, Wancheng Town, Wanning City; d Department of Gynecology, Hainan Modern Women & Infants Hospital, NO. 16 Jinyu East Road, Longhua District, Haikou City; e Department of Obstetrics; f Department of Obstetrics, People's Hospital of Wanning, No. 1 Huanshi 3rd East Road, Wancheng Town, Wanning City, Hainan Province, PR China.

**Keywords:** drospirone ethinyl estradiol, ethinyl estradiol cyproterone, polycystic ovarian syndrome, hypersotrophicemia, insulin resistance, meta-analysis

## Abstract

**Background:**

Polycystic ovarian syndrome (PCOS) is an endocrine disorder syndrome with reproductive dysfunction and abnormal glucose metabolism. Persistent non-ovulation, excessive androgens and insulin resistance are important features and they are the most common causes of menstrual disorders in women during childbearing years. At present, the cause of PCOS is not clinically clear. Current studies suggest that it may be due to the interaction of certain genetic genes with environmental factors. It is an important cause of infertility or early miscarriage with the characteristics of various causes and complex clinical manifestations. At present, for the treatment of PCOS patients, clinical treatment mainly includes hypoglycemia, insulin and menstrual regulation and other symptomatic and supportive treatment. Drospirone ethinyl estradiol and ethinyl estradiol cyproterone are 2 of the most commonly used drugs in clinical treatment of PCOS, but there is lack of the evidence of evidence-based medicine. Therefore, this study systematically evaluates the therapeutic effect and safety of PCOS patients with 2 short-acting oral contraceptives, drospirone ethinyl estradiol and ethinyl estradiol cyproterone, which provides the guidance for clinically selecting the appropriate drug to treat PCOS.

**Methods:**

Searching CNKI, WanFang Data, VIP, SinoMed, PubMed, EMbase, Web of Science, and The Cochrane Library database by computer, collecting the randomized controlled studies of DEE and EEC in the treatment of PCOS. The retrieval time limit is from the establishment of each database to July 1, 2020. In addition, tracing the references incorporated into the literature to supplement to the relevant literature. Using the retrieval method by combining the free words and the subject words, and the individual search of different databases is carried out. Meta-analysis is performed using RevMan 5.3 software after 2 researchers independently screens the literature, extracts the data, and evaluates the bias risk included in the study.

**Results:**

This study will systematically evaluate the DEE and EEC in the treatment of PCOS by collecting the required evidence to understand the effects of the 2 drugs on hypersotrophicemia, insulin resistance, lipid metabolism, and the safety during drug use in patients of this class, and the results will be published in highly influential academic journals.

**Conclusion:**

The results of this study will provide theoretical basis for the drug treatment of polycystic ovarian syndrome and provide help in the decision-making of clinical treatment of the disease.

**Ethics and dissemination:**

In this study, meta-analysis was used to conduct a second study on the published literature. Therefore, this type of systematic review research does not need to be approved by ethics.

**OSF Registration DOI:**

10.17605/OSF.IO/8GW9M.

## 1 Introduction

Polycystic ovarian syndrome (PCOS) is a more common endocrine disease in gynecological clinics, which is a very complex endocrine system disorder disease affected by a variety of factors.^[1]^ At the same time, PCOS is also a research focus and hot spot in the field of clinical reproductive medicine, and the incidence of the disease has been increasing year by year since the beginning of the 21st century.^[2]^ PCOS has a certain stage of onset, and the incidence rate of women in normal childbearing age is higher than that of other female groups. Clinically, it is characterized by persistent non-ovulation, bio-chemical or clinical manifestations of high androgens, and ovarian polycystic changes, and it often accompanied by obesity and insulin resistance.^[3–4]^ At present, the pathogenesis of PCOS is not completely clear, some studies believe that genetic factors, the abnormalities of hypothyroidism-pituitary-ovary axis regulation, adrenal endocrine mechanism disorders, ovarian local side secretion and self-secretion regulation mechanism imbalance, hypersotrophicemia and insulin resistance, the imbalance between inflammation factors and anti-inflammatory factors may lead to the occurrence of the disease.^[5–6]^

PCOS not only affects the ovulation function of patients, resulting in infertility. In the patients with long-term illness also find insulin resistance, glycolipid metabolism disorder, leading to the incidence rate of long-term complications such as type 2 diabetes, cardiovascular disease, endometrial cancer, ovarian cancer is increasing, and it brings great risks to the patients health and safety.^[7]^ The studies have found that nearly 70% of PCOS patients have insulin resistance. Hypersotrophicemia and insulin resistance play a key role in the development of this type of patients with disordered blood lipid levels. The common types of lipid metabolic imbalances in PCOS patients ae: elevation of total cholesterol, triglycerides, LDL-cholesterol, the levels of VLDL-C and apolipoprotein B increased, and the levels of HDL-C and apolipoprotein A1 decreased. The studies have confirmed that abnormal levels of these lipid indicators may increase the risk of atherosclerosis and coronary heart disease.^[8–9]^ Therefore, PCOS patients should take active treatment measures to prevent the cardiovascular disease and other long-term complications.

At present, clinical treatment methods mainly include lifestyle adjustment, drug treatment and surgical treatment.

1. lifestyle intervention: because there is a great relationship between the incidence of PCOS patients and obesity and environmental factors, it is important to adjust the lifestyle of PCOS patients to improve the clinical symptoms of patients. For example: reducing the intake of sugar and fat, smoking ban, alcohol ban, strengthening exercise, controlling the blood lipids, increasing the sensitivity of PCOS patients to insulin, thereby reducing the bodys insulin resistance, inhibiting the body to produce excessive levels of male hormones. At the same time, it should adjust the menstrual cycle of PCOS patients to promote the normal ovulation of patients and create a better fertilization environment and conditions for PCOS patients. It also reduces the risk of secondary cardiovascular disease and atherosclerosis.^[10–11]^
2. drug treatment: oral contraceptives, progesterone therapy, estrogen prologue therapy, ovulation-promoting therapy and insulin resistance therapy.^[12–13]^
3. surgical treatment: in recent years, the clinical use of surgical methods to treat PCOS patients has been rare, and it only provide for urgent fertility requirements and active requirements of surgery patients. In the months after surgery, local asosteroid status improves due to the loss of follicle fluid, which makes spontaneous ovulation possible after surgery.

But in about half of a year after the operation, the ovary local high hormone state is restored to preoperative levels again, again hindering the ovulation. In view of the limited effectiveness of surgical treatment, surgical treatment is not advocated as a priority treatment at present.^[14]^

Hormonal contraceptives are the first choice for the treatment of menstrual abnormalities, hirsutism and acne in PCOS patients, which can reduce luteinizing hormone (LH), testosterone levels and improve clinical symptoms such as hirsutism and acne. Drospirone ethinyl estradiol and ethinyl estradiol cyproterone are the 2 most commonly used drugs in clinical treatment of PCOS. However, the clinical studies have found that in normal women who have long chosen oral contraceptives as the primary method of contraception, blood lipid disorders have occurred, increasing the incidence of type 2 diabetes, cardiovascular diseases, and adversely affecting the prevention and treatment of complications such as long-term cardiovascular diseases.^[15]^ Therefore, this study systematically evaluates the therapeutic effect and safety of PCOS patients with 2 short-acting oral contraceptives, drospirone ethinyl estradiol and ethinyl estradiol cyproterone, in order to understand the effects of the 2 drugs on the patients with hypersotrophicemia, membrane resistance, lipid metabolism, and the safety of drug use, thus helping to choose the appropriate drug treatment for PCOS clinically.

## 2 Methods

1. Registered organization information: Open Science Framework, OSF.
2. Registration number: DOI 10.17605/OSF.IO/8GW9M
3. URL link for this study: https://osf.io/8gw9m.

### 2.1 Eligibility criteria

#### 2.1.1 Types of studies

A randomized controlled study of DEE and EEC in the treatment of PCOS is collected by searching the online electronic literature database, the scope of the literature is limited to studies published in Chinese and English.

#### 2.1.2 Types of participant

After diagnosis, the patient is diagnosed with PCOS (age, course of disease, complications and other conditions are not limited), which met the inclusion and exclusion criteria. Before the start of the study, no drugs interfering with the study ae taken.

#### 2.1.3 Inclusion criteria

In line with the Rotterdam standard proposed by ESHRE/ASRM in 2003.^[16]^

1. Rare ovulation or no ovulation (excluding non-ovulation caused by hyper-oxytocinemia, thyroid disease, etc.);
2. Clinical manifestations of asosteroids and hypersotrophicemia;
3. Ovarian polycystic changes: ultrasonography reveals that one or both ovaries has an inner diameter of 2–9 mm follicles ≧12, and ovarian volume ≧10 ml (ovarian volume = 0.5 × long diameter × transverse diameter × anterior and posterior diameter).

It can be diagnosed as polycystic ovarian syndrome after meeting 2 of the above 3 items and excluding other causes that may cause asosteroids.

#### 2.1.4 Exclusion criteria

1. Non-randomized controlled studies;
2. The articles without included outcome indicators or without access to the full text and raw data;
3. The study subject combined thyroid, adrenal diseases, abnormal liver and kidney function or other serious diseases;
4. In the last 3 months, sex hormone drugs have been used in therapy or in the course of treatment of combined use of other hormone drugs.

#### 2.1.5 Intervention measures

Control group: drospirone ethinyl estradiol + basic treatment (the treatment of lifestyle intervention + insulin resistance); Experiment group: ethinyl estradiol cyproterone + basic treatment (the treatment of lifestyle intervention + insulin resistance).

### 2.2 Types of outcome measures

#### 2.2.1 Main outcomes indicators

1. Clinical symptom improvement indicators: body mass index (BMI), waist to hip ratio (WHR), Ferriman-Galllwey score, acne score, systolic blood pressure (SBP), diastolic blood pressure (DBP).

2. Hormone Indicators: testosterone (T), sex hormone binding globulin (SHBG), free androgen index (FAI, FAI = T × 100/SHBG), luteinizing hormone (LH), follicle stimulating hormone (FSH).

#### 2.2.2 Additional outcomes indicators

1. Menstrual cycle.
2. Adverse reactions, observation of patients during treatment of digestive tract reactions, nervous system reactions, breast swelling pain, emotional abnormalities and other adverse reactions occurred.
3. Total cost of treatment.

### 2.3 Search strategy

By means of computer retrieval and manual supplementary retrieval, and comprehensively collecting the randomized controlled research literature of DEE and EEC in the treatment of PCOS for nearly 20 years, and using computers to retrieve the full-text database of CNKI, SinoMed, VIP, the WANGFANG Database, Web of Science, PubMed, the Cochrane Library and EMbase. Using medical subject words (MeSH) and index descriptors to optimize data searching. It also tracks the references included in the literature and the relevant systematic evaluation and summary literature. The retrieval period is from May 2000 to May 2020. The scope of the literature is limited to studies published in Chinese and English. Now the PubMed database, one of the English databases, is shown as an example of retrieval, see Table 1.

**Table 1:** Using PubMed database to show retrieval strategy.

Number	Search items
1	Drospirenone and ethinyl estradiol combination [mesh]
2	Cyproterone acetate, ethinyl estradiol drug combination [mesh]
3	Contraceptives, Oral [mesh]
4	Polycystic ovarian syndrome [mesh]
5	(Drospirenone, ethinyl estradiol drug combination) OR (YAZ combination) OR (yasminelle) OR (Yasmin) OR (Yaz) [tiab]
6	(Cyproterone acetate - ethinyl estradiol) OR (SHB 209 AE) OR (Diane-35) OR (Diane-35 Diario) [tiab]
7	(Oral Contraceptives) OR (Oral Contraceptives, Phasic) OR (Contraceptives, Phasic Oral) OR (Phasic Oral Contraceptives) OR (Oral Contraceptives, Low-Dose) OR (Oral Contraceptives, Low-Dose) OR (Low-Dose Oral Contraceptives) OR (Oral Contraceptives, Low Dose) OR (Contraceptive Agents, Female, Combined) OR (Oral Contraceptives, Combined) OR (Combined Oral Contraceptives) OR (Contraceptives, Combined Oral) [tiab]
8	(Ovary Syndrome, Polycystic) OR (Syndrome, Polycystic Ovary) OR (Stein-Leventhal Syndrome) OR (Stein Leventhal Syndrome) OR (Syndrome, Stein-Leventhal) OR (Sclerocystic Ovarian Degeneration) OR (Ovarian Degeneration, Sclerocystic) OR (Sclerocystic Ovary Syndrome) OR (Polycystic Ovarian Syndrome) OR (Ovarian Syndrome, Polycystic) OR (Polycystic Ovary Syndrome 1) OR (Sclerocystic Ovaries) OR (Ovary, Sclerocystic) OR (Sclerocystic Ovary) [tiab]
9	(Randomized Controlled Trial) [tiab]
10	1 OR 5 OR 3 OR 7
11	2 OR 6 OR 3 OR 7
12	4 AND 8
13	(10 AND 11) AND #12 AND #9

Table 1. Using PubMed database to show retrieval strategy.

### 2.4 Literature screening and data extraction

#### 2.4.1 Literature screening

**Figure 1:**
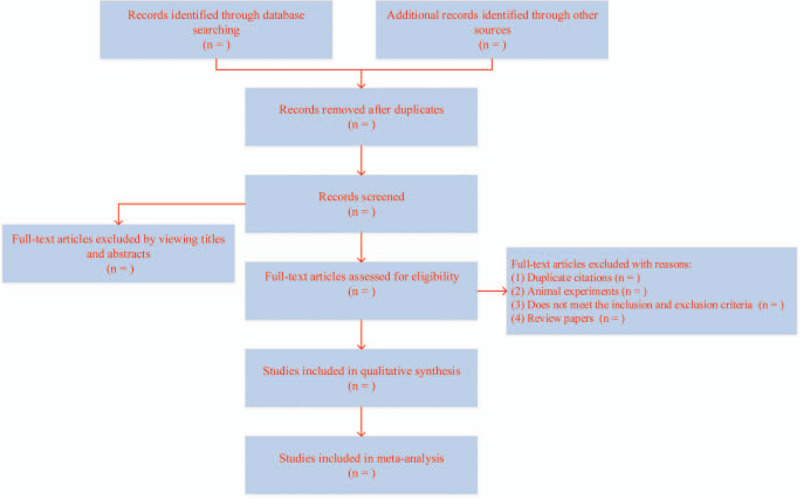
Flow chart of literature collection, elimination of duplicate documents and exclusion.

Two researchers independently carry out literature searching, and import the retrieved literature information into Endnote software, remove the duplicate literature; according to the inclusion and exclusion criteria screening literature. Figure 1 shows the complete process of the above steps. First reading the topic and abstract, removing the literature that does not meet the standards, the full text of the eligible documents is read by 2 evaluators after downloading it, if there are differences between the 2 evaluators, the third evaluator to discuss and decide whether to include. For documents that do not have access to data, it can contact the author by e-mail, telephone, etc., to obtain the data.

#### 2.4.2 Data extraction

The extracted content includes the following:

1. the basic situation and characteristics of the study, including the author, publication date, the type, sample content, intervention, control group situation, intervention cycle, frequency of intervention, evaluation tool of outcome indicators, results data of study;
2. indicators related to the evaluation of literature quality: the method of producing random sequences, the hiding of sample distribution sequences, the implementation of blind methods, whether the outcome indicators are complete, etc.

Any differences and uncertainties will be resolved by consensus between the 2 researchers or by asking a third researcher to make a final decision.

### 2.5 Literature quality assessment

According to the literature quality evaluation method recommended by Cochrane System Reviewer Manual 5.1.0, the quality of the included literature is evaluated, including random sequence generation, allocation of hidden program, blind methods of study subjects and interventionists, as well as outcome evaluators, integrity of outcome indicators, selective reporting results and other potential bias risks are evaluated, and the level of bias risk is judged according to specific evaluation criteria according to the requirements of the above 7 aspects. “Yes” represents low bias risk, and “Unclear” represents lack of relevant information or uncertainty about bias, “No” represents a high bias risk. The literature quality evaluation is carried out independently by 2 researchers and cross-checked. In case of disagreement, it is up to the third researcher to decide.^[17]^

### 2.6 Statistical analysis

Since the data differences in each included study are persistent, the heterogeneity existing between the data consolidation effect studies is absolute, so the information of the combined data must have better homogeneity in order to carry out the corresponding meta-analysis. The heterogeneity of the research results is described by the *P* value of the χ^2^ test and the heterogeneity index *I*^2^ value. Heterogeneity index *I*^2^ value is the minimum 0%, the value is up to 100%. When the heterogeneity index *I*^2^ = 0, there is no obvious heterogeneity, and when the heterogeneity index value is closer to 100%, the more obvious the heterogeneity is. According to the Coachrane System Evaluation Manual, the *P* value is 0.1, the value of I^2^ is bounded by 50%, and if the *P* value .1, *I*^2^ value <50% is that heterogeneity is not obvious, a fixed effect model can be used for meta-analysis. If the *P* value <.1, *I*^2^ value ≥50% indicates that heterogeneity is significant and that heterogeneity sources need to be analyzed and conducts meta-analysis by using random effect models. Moreover, when analyzing heterogeneity sources, secondary meta-analysis or sub-group analysis is usually carried out by excluding factors that may cause deviation in the analysis results, and by comparing the results of the 2 previous data analyses, it is necessary to decide whether sensitivity analysis is necessary. If heterogeneity is still significant after sensitivity analysis, especially when considering the possibility of methodological heterogeneity and clinical heterogeneity, meta-analysis needs to be abandoned and descriptive analysis based only on the results.

#### 2.6.1 Measures to deal with missing data

In the included study, if more than half of the data are missing or patients lost follow-up for more than 3 months, the data could not be included in the data integration analysis.

#### 2.6.2 Sensitivity analysis

Sensitivity analysis and heterogeneity test simultaneously analyze the sources of heterogeneity in the research literature, which is an important means to evaluate the stability of outcome indicators by using statistical test models. By replacing or changing one or more model types in the process of data analysis, the results are high stable and low sensitive if the results of the 2 sets of studies before and after the change are not significant. Sensitivity analysis in this meta-analysis uses the before and after replacement effect model to determine the stability of the outcome indicator by comparing the difference between the results of the 2 meta-analysis and the *P* value before and after.

### 2.7 Publication bias

Using the funnel plots drawn in the RevMan 5.3 software to assess whether the literature has publication bias, and drawing funnel plots requires that 7 pieces must be included in the study literature. If there is no publication bias in the funnel plots drawn by software, then each effect point in the figure should be evenly symmetrically distributed on both sides centered on the vertical axis (standard error), and the concentrated distribution at the tip of the funnel plot indicates that the sample size of the study data is large, while the distribution at the bottom of the funnel plot indicates that the sample size of the study data is small. If there is a significant asymmetry between the effect points in the funnel plot, it indicates that the study may have publication bias, the more significant the asymmetry, and the greater the degree of bias.

### 2.8 Evidence assessed

The evidence quality is evaluated by the recommended grading method of the GRADE system, and the evidence quality is divided into 4 levels, namely A (high quality), B (medium quality), C (low quality), and D (very low quality). The RCT study on the effectiveness and safety of drospirenone ethinyl estradiol and ethinyl estradiol cyproterone in the treatment of polycystic ovarian syndrome is evaluated for the evidence quality.^[18]^

## 3 Discussion

PCOS is one of the most common gynaecological endocrine diseases. PCOS patients with clinical manifestations and signs of high heterogeneity, mainly in the clinical manifestations of hypersotrophicemia and androgen excess (hair, acne, hair loss, etc.), insulin resistance and secondary high insulinemia, abnormal sugar tolerance, diabetes, obesity, metabolic syndrome, persistent non-ovulation or rare ovulation, infertility, endometrial lesions, etc., seriously affecting the physical and mental health of PCOS patients. Several big data studies have proved that the pathogenesis of PCOS is closely related to changes in the function of the bodys endocrine system. Because of the synthesis of LH and the increase in the magnitude and frequency of pulse secretion, to increase the levels of serum LH, while follicle-follicle-promoting hormone (FSH) secretion is normal or slightly reduced, high levels of LH can enable ovary interstitial and ovary membrane cells to multiply, resulting in increased androgen synthesis. It is not enough to reach the threshold of follicle collection to selection due to the low levels of FSH, so it cannot produce advantage follicles. Multiple follicles in the ovaries produce a large amount of estrogen, lack synergy with growth factors caused by the lack of dominant follicles, inhibit follicle apoptosis, and stagnate the development of sinus follicles in growth.^[19]^

PCOS is a disease that requires long-term treatment, there is no standard unified treatment plan, the course of treatment often takes more than 3 months, mainly to adjust the menstrual cycle and reduce androgen levels, patients with fertility requirements also need to induce ovulation. It can improve symptoms by adjusting the lifestyle, controlling diet, and increasing reasonable exercise to lose weight, while oral contraceptives can adjust the menstrual cycle in PCOS patients, reduce androgens, inhibit hair growth and treat acne, prevent endometrial lesions, and others. The drug drospirone ethinyl estradiol used in this study, each containing 30 μg ethinyl estradiol and drospirenone 3 mg, is the fourth generation of oral contraceptives.^[20–21]^ Drospirenone is a derivative of 17-α steroids, which is synthetic progesterones with natural progesterone active. In addition, it also has anti-androgens and anti-mineralocorticoid properties.

1. Drospirenone can feedback and inhibit the release of gonadotropins, lower the levels of LH, and inhibit the release of androgens from the ovarian and adrenal glands;
2. Although DRSP has a low affinity with androgen receptors, it can significantly increase the level of sex hormone binding globulin, so it can be used to treat hyperandrogenemia;
3. The ovarian contains renin-angiotensin system (RAS), which may cause an increase in androgens in the patients body.

There are expressions of renin and angiotensin II in the follicular membrane cells of the ovarian. In the local feedback loop of the ovarian, the increase of renin can lead to an increase in the content of angiotensin II, thereby stimulating the secretion of LH; renin can inhibit the aromatase activity of ovarian granulosa cells and hinder the transformation of androgen; and the levels of renin and angiotensin in this type of patients lose periodic fluctuations and continue to be at high levels, which stimulate the follicular membrane cells to produce androgens through paracrine methods. Drospirenone can resist mineralocorticoids, antagonize the RAS system, reduce the levels of renin and angiotensin, and improve the state of hyperandrogenemia; ethinyl estradiol cyproterone tablets are the third-generation oral contraceptives, each containing cyproterone 2 mg and ethinyl estradiol 0.035 mg.^[22–23]^ Containing cyproterone is a synthetic progesterone with high progesterone activity derived from 17-hydroxyprogesterone. It is an androgen antagonist that can inhibit the activity of P450c17/17–20 lyase and reduce androgen synthesis; it can be competitively combined the receptors of androgen and dihydrotestosterone antagonize androgen activity; it can reduce the level of androgens derived from the adrenal gland through hormone-like effects; at the same time, it can compete for androgen binding receptors on targeted organs and block the peripheral effects of androgens.

At present, drospirone ethinyl estradiol and ethinyl estradiol cyproterone as drugs for the treatment of polycystic ovarian syndrome has been widely used in clinical, but there is a lack of evidence-based medical evidence for the comparison of the treatment effectiveness and safety of these 2 drugs, so this study through meta-analysis methods, objectively evaluating the effectiveness of these 2 drugs to treat polycystic ovarian syndrome and the safety of drug during the use process, so as to provide guidance for clinicians in treatment decision-making.

## Author contributions

**Conceptualization:** Zhimin Liu, Ying Song, Yingchun Weng.

**Data curation:** Zhimin Liu, Ying Song.

**Formal analysis:** Zhimin Liu, Yuanfang Xu, Jing Wang.

**Methodology:** Zhimin Liu, Ying Song, Yuanfang Xu, Jing Wang, Hongyuan Hu.

**Project administration:** Zhimin Liu, Ying Song, Yingchun Weng.

**Resources:** Zhimin Liu, Yuanfang Xu, Hongyuan Hu.

**Software:** Ying Song, Jing Wang, Hongyuan Hu.

**Supervision:** Zhimin Liu, Yingchun Weng.

**Writing – original draft:** Zhimin Liu, Ying Song, Yuanfang Xu, Jing Wang, Hongyuan Hu.

**Writing – review & editing:** Yingchun Weng.
